# Strategies for Improving the Standardization of Perioperative Adverse Events in Surgery and Anesthesiology: “The Long Road from Assessment to Collection, Grading and Reporting”

**DOI:** 10.3390/jcm11175115

**Published:** 2022-08-30

**Authors:** Aref S. Sayegh, Michael Eppler, Jorge Ballon, Sij Hemal, Mitchell Goldenberg, Rene Sotelo, Giovanni E. Cacciamani

**Affiliations:** The Catherine and Joseph Aresty Department of Urology, USC Institute of Urology, Keck School of Medicine, University of Southern California, Los Angeles, CA 90033, USA

The assessment, collection, and reporting of all aspects of surgical procedures are crucial for optimizing patient safety and improving surgical/procedural quality [1]. Choosing the appropriate outcome reporting system is critical in order to achieve these goals and doing so successfully has the potential to foster a positive culture of adverse event (AE) reporting and accurate data comparison. Ultimately, such a collaboration between the surgical community could translate into improved patient outcomes by precisely capturing surgical events that lead to patient harm and develop measures to prevent them from happening [2,3,4].

Reporting guidelines are essential to enhance evidence-based research by standardizing information included in manuscripts, Thus ensuring the research is understood, replicable, and valuable for clinical decision-making. Reporting guidelines aid researchers, editors, and reviewers in maximizing the quality and reporting of scientific research [5] with the ultimate potential to increase publication citations and improve patient outcomes. Despite the availability of a variety of reporting guidelines, their usage remains subop-timal in the surgical literature [6].

Surgical and anesthesiologic outcomes must be reported consistently with clarity and transparency [7]. To achieve this, it is imperative to collect surgical and anesthesiologic outcomes routinely albeit with the necessary tools to identify and measure patient outcomes.

Reporting outcomes alone does not capture the entirety of the surgical procedure. A negative outcome (i.e., complication) could be secondary to AEs that occur in or around the operating room during the surgery. Unfortunately, there are no agreed-upon stand-ardized guidelines for AE assessment, collection, and reporting. These limitations con-tribute to the inconsistency and lack of clarity in studying and reporting surgical outcomes. Incomplete medical records, heterogeneous definitions of intraoperative variables, and retrospective data collection hinder the accurate portrayal of perioperative events [4,7]. Therefore, in order to ensure proper outcome reporting, there is a clear need for stand-ardization of accurate perioperative data collection (Figure 1). 

Perioperative data collection can be done prospectively or retrospectively. When collected retrospectively, data is retrieved from patient charts based on established protocols likely designed to fit the study’s agenda. Usually the retrospectively analyzed data was not intentionally collected for the purpose of a study, pertinent patient and intraoperative information is likely to be left out [8]. Thus, if appropriate tools are developed to collect standardized surgical data prospectively, it will simplify and expand on the information to study perioperative AEs and outcomes. 

We are not the first group to recommend standardization of the collection and reporting of surgery-related AEs. Martin et al. [7] proposed ten criteria to report complications following surgery; this proposal has been cited 675 times in the scientific literature. Mitropoulos et al. [9] built on Martin’s work, providing 13 criteria to improve the quality and accuracy of surgical data collection (cited 465 times) (Figure 2). Figure 2 reveals an unsurprising trend: a new tool will overtake its predecessor if it is of higher quality.

Similarly, the classification of intraoperative adverse events (iAEs) has been attempted [12,13,14,15], however, its use has not been broadly adopted. Consequently, iAE reporting remains heterogeneous, lacking consistency and comparability, and is rarely cited in the literature. iAE grading and reporting tools exist but are significantly underreported, limiting the quality of data that could be used to study their association with surgical outcomes [4,16,17,18].

In order to solve this gap and to respond to the urgent call for an impending need to adopt a standardized recording of perioperative outcomes, it is paramount to set a standardized protocol to facilitate the assessment, collection, and reporting of any aspect of an iAE that may contribute to the overall patient outcome. Furthermore, a standardized system will aid in limiting the spread of misinformation and underestimation of these events. We need a validated system maximized for simplicity (i.e., only capturing the minimum patient data needed for analysis), with specific guidelines (i.e., easily completed patient forms) to follow accurately assess, collect, grade, and report perioperative events. This type of system is not solely for prospective study purposes but can also be applied retrospectively to further our understanding as a community of AEs that lead to patient harm with the ultimate goal of reducing preventable AEs [19]. Thus, outcome-related data collection may directly improve patient outcomes, in addition to producing a wealth of information that could be used to indirectly improve patient outcomes.

Properly reporting AEs in academic literature is essential for understanding the true incidence and impact of these events on patient safety and outcomes. Therefore, the Intraoperative Complication Assessment and Reporting with Universal Standards (ICARUS) Global Surgical Collaboration is working on providing the resources needed to accelerate the worldwide adoption and rigorous implementation of a culture of assessment, collection, grading, and reporting of iAEs [2,3,4,20,21,22]. Such collaboration should increase the quality of research, which in turn has lasting implications for how we approach medical academia and surgical and anesthesiologic care for patients. Accurate reporting is an essential component of high-quality research and responsible physician and surgeon.

## Figures and Tables

**Figure 1:**
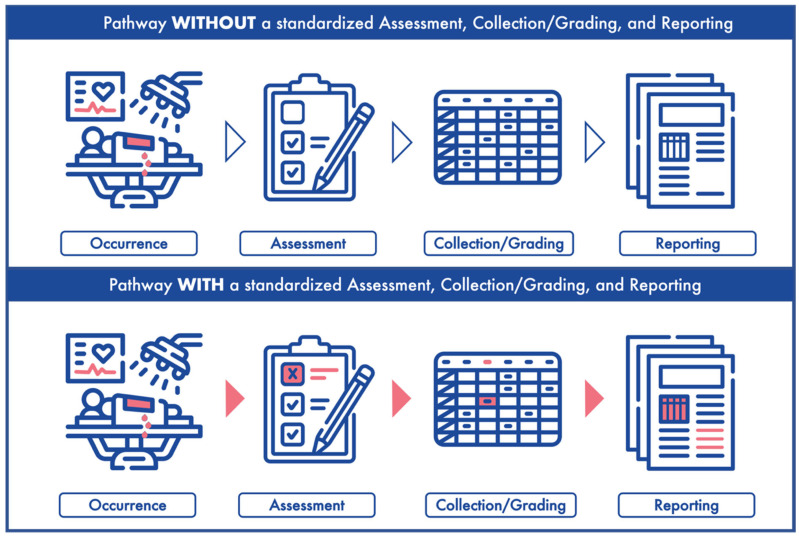
**The long road from occurrence to reporting.** (**Upper panel**) depicts an adverse event occurrence, assessment, collection/grading and reporting without a standardized system. (**Lower panel**) represents an adverse event occurrence, assessment, collection/grading and reporting with a standardized system.

**Figure 2:**
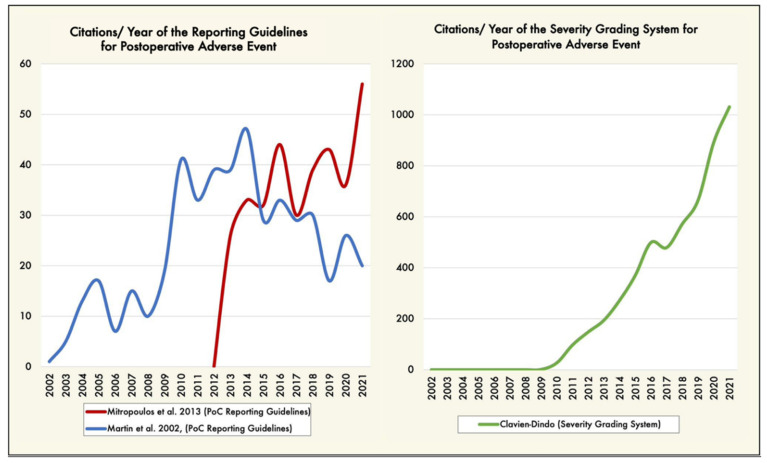
(**Left graphic**) Trends of articles citing the postoperative adverse event reporting guidelines [7,9], by year (blue and red lines). (**Right graphic**) Trends of articles reporting postoperative complications severity grading system [10,11], by year (green line). Trends over time were performed using the Web of Science citation database (https://tinyurl.com/3emtzjw8 accessed on 17 August 2022, date last accessed). PoC: postoperative complications.

## References

[1] Mitropoulos D., Artibani W., Biyani C.S., Jensen J.B., Remzi M., Roupret M., Truss M. (2014). Quality assessment of partial nephrectomy complications reporting using EAU standardised quality criteria. Eur. Urol..

[2] Cacciamani G.E. (2022). Intraoperative adverse events grading tools and their role in honest and accurate reporting of surgical outcomes. Surgery.

[3] Cacciamani G.E., Sholklapper T., Dell’Oglio P., Rocco B., Annino F., Antonelli A., Amenta M., Borghesi M., Bove P., Bozzini G. (2022). The Intraoperative Complications Assessment and Reporting with Universal Standards (ICARUS) global surgical collaboration project: Development of criteria for reporting adverse events during surgical procedures and evaluating their impact on the postoperative course. Eur. Urol. Focus.

[4] Cacciamani G.E., Sholklapper T., Dell-Kuster S., Biyani S.C., Francis N., Kaafarani H.M., Desai M., Gill I., Collaboration I.G.S. (2022). Standardizing The Intraoperative Adverse Events Assessment to Create a Positive Culture of Reporting Errors in Surgery and Anesthesiology. Ann. Surg..

[5] Hope D.L., King M.A. (2022). The ‘so what’of reporting guidelines. Int. J. Pharm. Pract..

[6] Jin Y., Sanger N., Shams I., Luo C., Shahid H., Li G., Bhatt M., Zielinski L., Bantoto B., Wang M. (2018). Does the medical literature remain inadequately described despite having reporting guidelines for 21 years?—A systematic review of reviews: An update. J. Multidiscip. Healthc..

[7] Martin R.C., Brennan M.F., Jaques D.P. (2002). Quality of complication reporting in the surgical literature. Ann. Surg..

[8] Talari K., Goyal M. (2020). Retrospective studies–utility and caveats. J. R. Coll. Physicians Edinb..

[9] Mitropoulos D., Artibani W., Graefen M., Remzi M., Roupret M., Truss M. (2012). Reporting and grading of complications after urologic surgical procedures: An ad hoc EAU guidelines panel assessment and recommendations. Eur. Urol..

[10] Dindo D., Demartines N., Clavien P.-A. (2004). Classification of surgical complications: A new proposal with evaluation in a cohort of 6336 patients and results of a survey. Ann. Surg..

[11] Clavien P.A., Barkun J., De Oliveira M.L., Vauthey J.N., Dindo D., Schulick R.D., De Santibañes E., Pekolj J., Slankamenac K., Bassi C. (2009). The Clavien-Dindo classification of surgical complications: Five-year experience. Ann. Surg..

[12] Dell-Kuster S., Gomes N.V., Gawria L., Aghlmandi S., Aduse-Poku M., Bissett I., Blanc C., Brandt C., Ten Broek R.B., Bruppacher H.R. (2020). Prospective validation of classification of intraoperative adverse events (ClassIntra): International, multicentre cohort study. BMJ.

[13] Kaafarani H.M., Mavros M.N., Hwabejire J., Fagenholz P., Yeh D.D., Demoya M., King D.R., Alam H.B., Chang Y., Hutter M. (2014). Derivation and validation of a novel severity classification for intraoperative adverse events. J. Am. Coll. Surg..

[14] Biyani C.S., Pecanka J., Rouprêt M., Jensen J.B., Mitropoulos D. (2020). Intraoperative adverse incident classification (EAUiaiC) by the European Association of Urology ad hoc complications guidelines panel. Eur. Urol..

[15] Francis N., Curtis N., Conti J., Foster J., Bonjer H., Hanna G. (2018). EAES classification of intraoperative adverse events in laparoscopic surgery. Surg. Endosc..

[16] Cacciamani G.E., Maas M., Nassiri N., Ortega D., Gill K., Dell’Oglio P., Thalmann G.N., Heidenreich A., Eastham J.A., Evans C.P. (2021). Impact of pelvic lymph node dissection and its extent on perioperative morbidity in patients undergoing radical prostatectomy for prostate cancer: A comprehensive systematic review and meta-analysis. Eur. Urol. Oncol..

[17] Cacciamani G.E., Tafuri A., Iwata A., Iwata T., Medina L., Gill K., Nassiri N., Yip W., de Castro Abreu A., Gill I. (2020). Quality assessment of intraoperative adverse event reporting during 29 227 robotic partial nephrectomies: A systematic review and cumulative analysis. Eur. Urol. Oncol..

[18] Dell’Oglio P., Andras I., Ortega D., Galfano A., Artibani W., Autorino R., Mazzone E., Crisan N., Bocciardi A.M., Sanchez-Salas R. (2021). Impact of the implementation of the EAU guidelines recommendation on reporting and grading of complications in patients undergoing robot-assisted radical cystectomy: A systematic review. Eur. Urol..

[19] Wanderer J.P., Gratch D.M., Jacques P.S., Rodriquez L.I., Epstein R.H. (2018). Trends in the prevalence of intraoperative adverse events at two academic hospitals after implementation of a mandatory reporting system. Anesth. Analg..

[20] Cacciamani G., Sholklapper T., Sotelo R., Desai M., Gill I. (2021). A protocol for the development of the intraoperative complications assessment and reporting with universal standards criteria: The ICARUS Project. Int. J. Surg. Protoc..

[21] Cacciamani G., Sholklapper T., Dell-Kuster S., Biyani C., Francis N., Kaafarani H., Desai M., Sotelo R., Gill I. (2022). Assessing, grading, and reporting intraoperative adverse events during and after surgery. Br. J. Surg..

[22] Sholklapper T., Goldenberg M., Lebastchi A., Abreu A., Desai M., Sotelo R., Gill I., Cacciamani G. (2022). A1060—Intraoperative adverse event reporting in urology: Global ICARUS survey results. Eur. Urol..

